# Swift and Complete Healing of Digital Ulcers after Macitentan Treatment

**DOI:** 10.1155/2016/1718309

**Published:** 2016-11-22

**Authors:** Emilio Giner Serret

**Affiliations:** Servicio de Reumatología, Hospital Obispo Polanco, Teruel, Spain

## Abstract

Digital ulcers are a burdensome and painful condition with sparse options of treatment. We report the case of a 78-year-old female patient with limited cutaneous systemic sclerosis that sequentially developed digital ulcers. After the appearance of digital ulcers in the soles of her feet she was successfully treated with bosentan. The report of two new digital ulcers in her hands 9 months later alongside with elevated transaminase levels led to a switch to macitentan treatment. A swift and complete healing of both digital ulcers was observed after 3 months, with the restoration of normal biochemical values.

## 1. Introduction

Systemic sclerosis (SSc) is a complex multisystem disease characterized by dysregulation of the immune system associated with the presence of selective autoantibodies, vascular involvement, and multiorgan fibrosis [1]. Different factors, including genetic, environmental, vascular, autoimmunologic, and microchimeric factors, are involved in systemic sclerosis pathogenesis [2]. This is a rare disease, with an estimated prevalence in northwestern Spain of 27.7 cases per 100,000 people [3], being limited cutaneous SSc (62% of patients) the most common subset of patients [4].

Digital ulcers (DU) are a common and serious clinical manifestation of SSc (affecting approximately 40% of patients) [4], typically occurring on the fingertips. Digital ulcers are the consequence of an ischemic injury leading to necrosis of skin and subcutaneous tissues. The presence of DU is associated with increased burden and disability, reduced quality of life, impairment of daily activities, and decreased survival [5–7].

The current options of treatment for DU are scarce, and its experience of use is mainly limited to small studies and case series. Macitentan, a novel tissue-specific dual endothelin (ET) receptor antagonist, failed to reduce the frequency of new DU in two randomized trials [8] but, similar to bosentan (another dual ET receptor antagonist), it may be a rapid and effective option for the treatment of active DU in selected SSc patients.

## 2. Case Presentation

The patient was first referred to our service in April 1998 (woman; age: 60 years; current age: 78 years). At that moment we diagnosed her with limited cutaneous SSc according to the following manifestations: Raynaud's phenomenon (RP); sclerodactyly; anti-nuclear antibodies (ANA) titre: 1 : 1280 with speckled pattern; anti-Ro: positive; anti-centromere: positive; and enlarged capillaries in nailfold capillaroscopy.

She also had a medical history of primary Sjögren syndrome, stage III sarcoidosis (with bilateral interstitial infiltrates), and dyslipidemia. The patient was initially treated with nifedipine 20 mg retard for her RP, switching to diltiazem 120 mg retard after being hospitalized due to atrial fibrillation (September 2013; she started acenocoumarol). Follow-up visits were scheduled every 16 weeks thereafter, maintaining RP clinical control.

Eighteen months ago (February 2015) she reported two DU (one in each sole of her feet) and was prescribed oral bosentan 62.5 mg twice daily during the first month and 125 mg twice daily thereafter. After 3 months of bosentan treatment (May 2015) she completely healed of her initial DU, without presenting additional DU.

**Figure 1:**
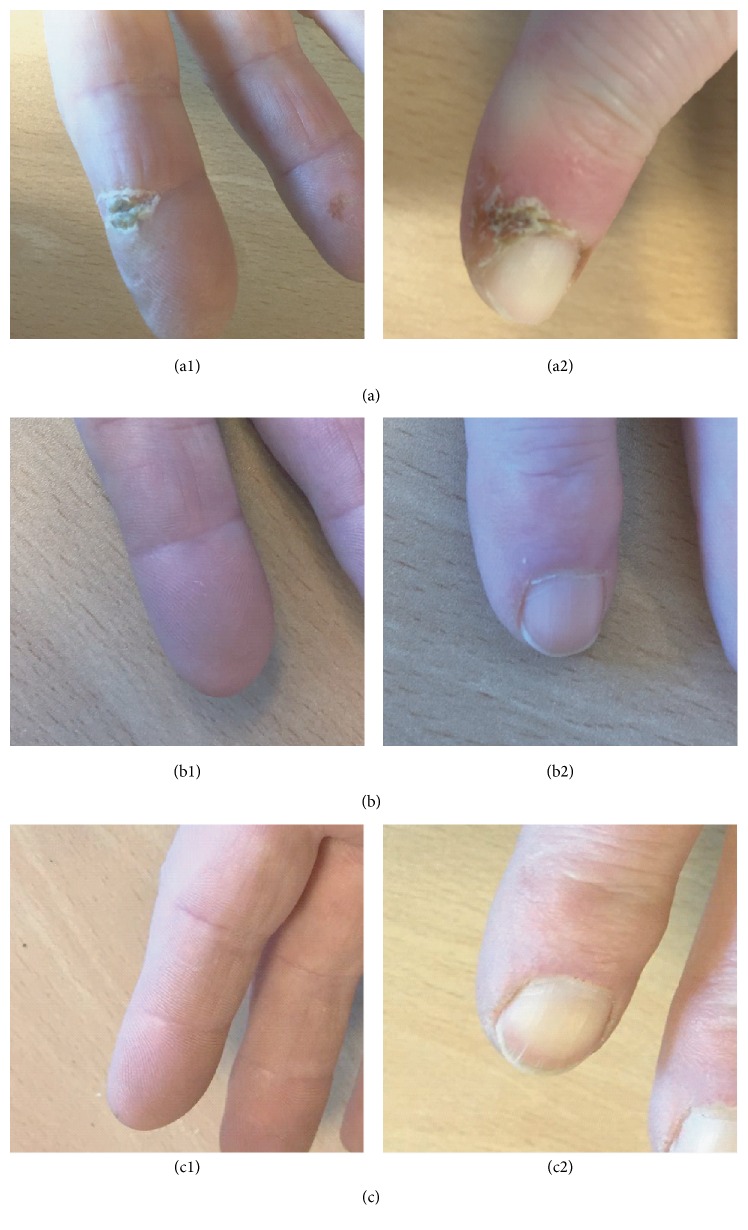
Evolution of digital ulcers at (a1, b1, and c1) second distal interphalangeal joint of the right hand and (a2, b2, and c2) second distal phalanx of the left hand at (a) baseline (before treatment with macitentan); (b) 3 months after treatment with macitentan; (c) 6 months after treatment with macitentan.

On November 2015 she came to our office after the sudden appearance of DU in her second distal interphalangeal joint (palmar) of the right hand and second distal phalanx of the left hand (dorsal) (Figure 1(a)). The laboratory tests also showed a hypertransaminasemia (aspartate aminotransferase (AST): 73 U/L; alanine transaminase (ALT): 90 U/L; alkaline phosphatase (ALP): 161 U/L; and gamma-glutamyl transferase (GGT): 208 U/L).

Due to this DU worsening and the elevated values of transaminases secondary to bosentan, the treatment was switched to macitentan 10 mg once daily (bosentan was titrated to 62.5 mg BID until macitentan became approved and available at our hospital). No concomitant treatments were administered.

The patient started macitentan treatment by December 15, 2015. After 3 months of treatment (February 2016) the patient rapidly reduced her DU (Figure 1(b)) and controlled her transaminase levels (AST: 20 U/L; ALT: 23 U/L; ALP: 120 U/L; GGT: 29 U/L).

After 6 months of treatment (June 2016), the DU remained completely healed (Figure 1(c)) with no appearance of new DU. The patient is still receiving 10 mg of macitentan once daily. Outpatient follow-up of the patient continues.

## 3. Discussion

To the best of our knowledge this is the first reported case of a SSc patient treated with macitentan achieving complete DU healing. In vitro studies have shown that ET is released by scleroderma fibroblasts, suggesting its pathogenic pathway in scleroderma. Macitentan acts as a dual ET receptor antagonist disrupting the vasoconstrictive and profibrotic effects of endothelin [9]. Compared with other ET receptor antagonists macitentan exhibited sustained receptor binding and enhanced tissue penetration [10]. Our results bolster the evidence regarding the role of ET in the vascular manifestations of SSc.

Currently bosentan (another ET receptor antagonist) is the only licensed treatment in Europe for preventing the appearance of new DU in SSc patients, based on two large randomized clinical trials [11, 12], but failed to confirm its efficacy in the healing of active digital ulcers. However, several additional publications suggest that bosentan may provide an effective and swift response in the treatment of digital ulcers [13–19], as observed in the case herein presented. Macitentan also provided rapid (less than 3 months) and complete healing while maintaining a normal hepatic function and without observed DU recurrence after 6 months, arguing in favor of its use. A longer follow-up period is required to confirm these findings.

Several other treatments have also been proven to be effective in small studies and case series, including prostanoids (iloprost [20] and epoprostenol [21]), calcium antagonists (nifedipine [22]), and PDE-5 inhibitors (sildenafil [23]), but this evidence is still limited. Accordingly, we encourage the report of any additional information that may shed more light on the treatment of DU in SSc patients given the impact of this disabling condition.

In summary, these results suggest that macitentan may promote the healing of active digital ulcers in certain patients with scleroderma. An extensive study aiming to characterize this selected population is desirable.
